# A cross-sectional serosurvey on hepatitis B vaccination uptake among adult patients from GP practices in a region of South-West Poland

**DOI:** 10.1186/s12889-015-2388-8

**Published:** 2015-10-16

**Authors:** Maria Ganczak, Gabriela Dmytrzyk-Daniłów, Marcin Korzeń, Zbigniew Szych

**Affiliations:** Department of Public Health, Pomeranian Medical University, Zolnierska 48, 71-210 Szczecin, Poland; Vaccination Unit, Primary Care Clinic WS SPZOZ, Warszawska 30, Zgorzelec, Poland; Faculty of Computer Science and Information Technology, West Pomeranian University of Technology, Zolnierska 49, 71-210 Szczecin, Poland; Department of Computer Science and Education Quality Research, Pomeranian Medical University, Zolnierska 53, 71-210 Szczecin, Poland

**Keywords:** HBV, Vaccination, Coverage, Determinants, Anti-HBs, Anti-HBc total

## Abstract

**Background:**

Hepatitis B is a significant health burden in Poland with nosocomial transmission being the main source of infection. Therefore, HBV vaccination is widely recommended for those not covered by the national immunisation program. Objective: To assess the coverage and influencing determinants of HBV vaccination among adult patients attending GP clinics as well as to establish serological status in terms of HBV infection.

**Methods:**

Patients who were seen consecutively in March 2013 at four randomly selected GP practices located in Zgorzelec county, in south-western part of Poland, were invited to participate and complete questionnaires on socio-demographic data and other factors related to vaccination. A pilot study was done in one urban GP practice in the city of Gryfino (Gryfino county), the results have been included in the study. Patients’ immunisation status was assessed basing on vaccination cards and anti-HBs titer with the use of third-generation testing methods. In addition, serum samples were assayed for anti-HBc total.

**Results:**

Response rate: 99.3 %. Of 410 participants (66.1 % females, median age 56 years), 55.4 % (95%CI:50.5-60.1 %) were previously vaccinated; in those 11.5 % took 2 doses, 66.1 % - 3 doses,18.1 % – 4 doses. Elective surgery was the main reason (57.7 %) for HBV immunization, 4.8 % - were vaccinated due to recommendations by GPs. The multivariable logistic regression model revealed that living in a city (OR 2.11), and having a surgery in the past (OR 2.73) were each associated with greater odds of being vaccinated. Anti-HBc total prevalence among those unvaccinated was 13.6 % (95%CI:9.3 %-19,5 %), and 7.2 % (95%CI:4.4-11.8 %) among those vaccinated.

**Conclusions:**

Low HBV immunization coverage among adult patients from GP clinics and the presence of serological markers of HBV infection among both - those unvaccinated and vaccinated call for comprehensive preventative measures against infection, including greater involvement of family doctors. Although interventions should cover the whole population, inhabitants living in the rural areas should be a group of special interest. Preoperative immunization for HBV seems to be an efficient public health tool to increase the vaccination uptake.

## Background

According to the most recent World Health Organization estimate, two billion people worldwide have been infected with hepatitis B virus (HBV), 240 million are chronically infected, and approximately 780,000 persons die each year from hepatitis B (HB) infection – 650,000 from cirrhosis and liver cancer due to chronic HB infection and another 130,000 from acute HB [1, 2]. Although Poland is classified as a geographic region of low endemicity of the prevalence of hepatitis B surface antigen (HBsAg), HB is still a significant health burden. There are approximately 350,000-450,000 carriers (1.5 %) of HBsAg in the population [3, 4].

In 1986 safe and effective DNA recombinant vaccines became available, which are now one of the most widely used worldwide [5]. In Poland, neonatal vaccination began in 1989 with neonates born to HBsAg positive mothers. From 1993 to 1995 it was gradually expanded to neonates from some selected regions with the highest incidence of HBV infection; then, since 1996 it has covered all neonates [6, 7]. Since that period, active immunisation is also offered to recipients of blood and blood products, hemodialysed patients, household members and sexual partners of HBsAg carriers, as well as health care workers and medical students [6, 7]. In 2000, universal vaccination of adolescents of 14 years of age was introduced for those not covered by neonatal vaccination [6].

Although HBV vaccination is clearly the most effective strategy for preventing HB and the vaccine has been available for almost 30 years, the extent to which such a strategy is practiced or how the society follows it at the primary care level have not been precisely evaluated not only in Poland, but also in many other European Union (EU) countries. None of the EU countries surveyed in the VENICE project between 2010 and 2011 was able to provide coverage estimates for HBV adult vaccine [8].

According to data from the 1970s to 1990s, about 60 % of HB adult cases in Poland were acquired as a result of hospitalization or medical procedures provided in other health care facilities [8]. Therefore, between 1993 and 1997, active immunization against HB with two doses of a vaccine was required from all elective surgery patients to reduce the number of infections generated nosocomially. It has been estimated that 681,000 patients were vaccinated in 1997 due to this regulation [9]. The immunization requirement for preoperative HBV vaccination no longer applies due to slow but steady improvements in aseptic conditions (e.g. in 1994 in Poland, there were 5000 autoclaves in hospitals and 60,000 dry heat sterilizers, i.e. a ratio of 1:12, by the end of 1997 it had fallen to 1:7.7, since the end of 2009 dry heat sterilizers are no longer used) [6] as well as an increase in disposable medical devices use. Another reason was a national discussion where the opponents argued that the preoperative HBV vaccination policy might be a stimulus for health care facilities to neglect infection control procedures [9].

However, currently, a preoperative immunisation for HBV is still recommended by the National Immunisation Program [10] and an immunisation certificate is unofficially required for elective surgical procedures in some health care facilities. Our previous study revealed that such a policy influenced the very high immunisation coverage (82 %) among surgical patients admitted for elective procedures [11]. However, it has not been assessed so far to which extent patients who were operated on after taking two doses of a HBV vaccine complete the 3-dose course in a relevant time.

### Objectives

The objective of the study was to assess the HBV vaccination uptake among adult patients from GP practices, to define the reasons for immunization, the contributing factors as well as to evaluate the prevalence of serological markers of HBV infection among this population.

## Methods

### Design & setting

A cross-sectional sero-epidemiological survey was conducted in March 2013 among patients presented at randomly selected GP practices from the county of Zgorzelec, Poland. Patients 27 years old and more were invited to participate, due to the fact that in Poland the universal vaccination of adolescents of 14 years of age was introduced in 2000 for those not covered by neonatal vaccination. Therefore, in 2013, when the participants of this study were recruited, the adult population less than 27 years of age was protected against HBV infection through universal immunization.

### Study population & sampling

The sampling frame included a list of GP clinics in the Zgorzelec county obtained from the local health department. All GP practices in the region were stratified into urban and rural, to ensure representation of different practice levels. Random selection of 3 urban practices and 1 rural practice was done, proportionally to the number of patients covered by each type of practice. A pilot study was done in one urban GP practice in the city of Gryfino (Gryfino county) on 107 patients [12], the results have been included in the study. The authors’ choice regarding the relatively numerous group of patients involved in the pilot study was due to the fact that it was the first sero-survey conducted in Poland which queried GP clinic patients on their vaccination status not only with the help of questionnaires, but also by obtaining their blood samples. Therefore, the authors’ intention was to check various possible technical problems connected with blood sample collection and management (i.e. connected with questionnaire administration in relation to blood collection, a person responsible for blood collection, the delivery of blood samples to the lab etc.), as well as conducting the study in a timely manner. The anonymity of the subjects was preserved by a code given to the patient, for the questionnaire and for the blood sample. At each facility blood samples were obtained from all eligible patients who gave informed written consent to participate.

### Study instrument

A questionnaire administered by one of the researchers (G.D-D.) included questions that queried patients on the following:

- ▪demographic including age, gender, residency, literacy, socio-economic status, employment status
- ▪facility location
- ▪history of HBV vaccination
- ▪reasons for HBV vaccination

In addition, knowledge about HBV infection was assessed by 22 questions grouped in 4 categories: natural history of HBV, transmission routes, immunization, treatment. The overall score for each respondent was calculated, with the maximum score 22 and minimum 0 points.

### Sero-testing

HBV immunization status was based on the results of vaccination cards and by the enzyme immunoassay for the quantitative detection of antibodies to the surface antigen of HBV (anti-HBs). Qualitative detection of total antibodies to the core antigen (anti-HBc total) was used (Hoffman-La Roche Ltd., Basel, Switzerland). After collecting blood samples by one of the researchers (G.D-D.), testing was performed in two reference laboratories affiliated to teaching hospitals which had accreditation in the field of immunoassay analyses: in Szczecin (pilot group) and in Wrocław (study group). Two weeks after sampling the participants could call the investigators at a dedicated phone line and obtain their results by stating their code. The study received ethical approval from the Dolnoslaskie Region Ethical Committee (1/DR/2013).

### Statistical analysis

Data analysis used the STATISTICA (PL Version 7.1., StatSoft Inc., 2005) and R (R version 3.0.2) software [13]. Our primary outcome variable was HBV vaccination and we aimed to identify variables associated with this outcome. Univariate analysis assessed demographic characteristics (age, gender, residency, literacy, socio-economic and employment status), together with knowledge on HB, undergoing surgery in the past, facility location (urban/rural), and GP clinic associated with an outcome variable. For categoric variables groups were compared using the chi square and Fisher's exact tests, whilst the U Mann–Whitney test was used for numeric variables. Variables whose p-values at the univariate level were lesser than 0.25 were used to build a multivariable regression model [14], with the help of R software [13]. Regression coefficient (beta) was used to assess a change in the model. Coefficients for binary variables are equal to the natural logarithm of the odds ratio; OR = exp(beta) [15].

## Results

Of the total 413 consecutive patients eligible (i.e. ≥ 27 years old), 410 (99.3 %) consented to participate, 271 of them (66.1 %) were females. The median age for the study population was 56 years (range 27–85). Almost two thirds (64.9 %; *n* = 266) of participants were from the urban areas, 35.1 % (*n* = 144) from the rural areas. More than one third of participants (37.1 %; *n* = 152) were high school graduates, 28.3 % (*n* = 116) had vocational education, 17.8 % (*n* = 73) had a university degree, 16.8 % (*n* = 69) had primary education. There were 13.4 % (*n* = 55) participants who described their socioeconomic status as high, 69.5 % (*n* = 285) - as moderate, 17.1 % (*n* = 70) as low. Almost a half of participants (47.1 %; *n* = 193) were employed, 31.7 % (*n* = 130) retired, 12.0 % (*n* = 49) unemployed. Most of the participants (81.7 %; *n* = 335) were from health-care facilities located in urban areas, 18.3 % (*n* = 75) from facilities located in rural areas (Table 1).

**Table 1:** Participant characteristics by GP clinic. Zgorzelec county, Poland, 2013; *n* = 410

Variable	GP clinic																			
	GP1 (urban)*				GP2 (urban)				GP3 (rural)				GP4 (urban)				GP5 (urban)			
	n_1_	n_2_	% n_1_	p**	n_1_	n_2_	% n_1_	p**	n_1_	n_2_	% n_1_	p**	n_1_	n_2_	% n_1_	p**	n_1_	n_2_	% n_1_	p**
Vaccination uptake (Yes/No)	60	47	56.1	1.0	50	27	64.9	0.06	46	29	61.3	0.25	34	41	45.3	0.07	37	39	48.7	0.25
Age (≤50/>50 years)	47	60	46.4	0.0002	32	45	41.6	0.04	24	51	32.0	0.18	26	49	34.7	0.71	19	57	25.0	0.0001
Gender (F/M)	79	28	73.8	0.06	47	30	61.0	0.35	51	24	68.0	0.79	49	26	65.3	0.89	45	31	59.2	0.18
Education (university/other)	15	92	14.0	0.19	18	59	23.4	0.10	14	61	18.7	0.92	11	64	14.7	0.43	15	61	19.7	0.66
Residency (urban/rural)	72	35	67.2	0.56	64	13	83.1	0.0002	4	71	5.3	<0.0001	66	9	88.0	<0.0001	60	16	78.9	0.005
Socio-economic status (high/low-median)	11	96	10.3	0.32	14	63	18.2	0.19	11	64	14.7	0.71	8	67	10.7	0.57	11	65	14.5	0.71
Employment (yes/no)	46	61	43.0	0.32	43	34	55.8	0.10	32	43	42.7	0.44	41	34	54.7	0.16	31	45	40.8	0.25
Past surgery (yes/no)	65	26	60.7	1.0	58	19	75.3	0.40	55	20	73.3	0.67	52	23	69.3	0.77	50	26	65.8	0.26
HBV knowledge (high/low)	30	77	28.0	<0.0001	32	45	41.6	0.31	44	31	58.7	0.03	42	33	56.0	0.10	44	32	57.9	0.04
Total	107				77				75				75				76			

*pilot group

**proportions in a selected GP clinic vs other GP clinics

As presented in Table 1, overall, only slight differences were observed between facilities regarding participant characteristics; it refers to age and HBV knowledge. Participants from the pilot group were significantly younger (*p* < 0.0002) and less knowledgeable (*p* < 0.0001) than those in the other GP clinics. Participants from the GP2-urban clinic were significantly younger (*p* < 0.04) than those in the other GP clinics. Participants from the GP3-rural clinic were more knowledgeable (*p* < 0.03) than those in the other GP clinics, the same refers to participants from the GP5-urban clinic (*p* < 0.04). There were statistically significant differences in the proportions of participants living in urban versus rural areas between clinics (*p* < 0.0001 - *p* = 0.005 respectively), except the pilot group in which such proportions did not differ when compared to other GP clinics (*p* = 0.56). There were not any statistically significant differences observed in vaccine uptake by clinic.

As there is a free choice concerning the type of facility, regardless the place of residence, a proportion (73/335; 21.8 %) of residents from the rural areas attended facilities in the urban areas and vice versa - a proportion (4/75; 5.3 %) of residents from the urban areas attended a facility in the rural area.

### HBV vaccine uptake

**Fig. 1:**
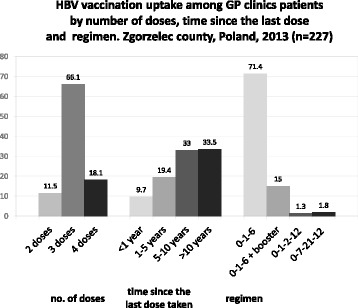
HBV vaccination *uptake among GP clinics patients by number* of doses*,* time since *the last dose and regimen.* Zgorzelec county, Poland, 2013 *(n = 227)*

Figure 1 presents HBV vaccine uptake by number of doses, time category since receiving most recent vaccine dose, and vaccination schedule. More than a half of the participants - 55.4 % (227/410; 95%CI:50.5-60.1 %) were previously vaccinated against HBV; in those 11.5 % (26/227; 95%CI:7.9-16.3 %) with 2 doses of vaccine, 66.1 % (150/227; 95%CI:59.7-71.9 %) with 3 doses, 18.1 % (41/227; 95%CI:13.6-23.6 %) with 4 doses, 4.4 % (10/227; 95%CI:2.4-7.9 %) did not remember the number of doses taken.

From 227 participants, 9.7 % (*n* = 22) were vaccinated against HBV 0–12 months ago, 19.4 % (*n* = 44) over 1 but less or equal to 5 years ago, 33.0 % (*n* = 75) over 5 but less or equal to 10 years ago, 33.5 % (*n* = 76) more than 10 years ago, 4.4 % (*n* = 10) did not remember and their vaccination card was missing (Fig. 1). HBV vaccination by numbers of doses received versus time category since receiving most recent vaccine dose is presented in Table 2.

**Table 2:** HBV vaccination among GP clinics patients: numbers of doses received versus time category since receiving most recent vaccine dose

No of doses received	No of participants	Time category since receiving most recent vaccine dose					
		0-5months		>5 months-1 year		>1 year	
		*n*	%	*n*	%	*n*	%
2	26	4	15.4	3	11.5	19	73.1
3	150	0	0	14	9.3	136	90.7
4	41	0	0	1	2.4	40	97.6
Total	217	4	1.8	18	8.3	195	89.9

Zgorzelec county, Poland, 2013

Most of the participants (71.4 %; 162/227) were vaccinated with the use of 0-1-6 regimen, 15.0 % (34/227) with a 0-1-6 regimen plus a booster dose, 1.3 % (3/227) with a 0-1-2-12 regimen, 1.8 % (4/227) with a 0-7-21-12 regimen, 10.5 % (24/227) did not remember the schedule (Fig. 1).

### Reasons for immunization

Participants gave reasons for immunization: 57.7 % (*n* = 131) were immunized due to the recommendation of surgeons referring them for various procedures, 10.1 % (*n* = 23) due to media campaigns, 4.8 % (*n* = 11) due to the recommendations of family doctors, 4.0 % (*n* = 9) due to the recommendations of family/friends, in 2.2 % (*n* = 5) of cases the decision was made due to a travel to countries of high or intermediate HBV endemicity, the rest (21.1 %; *n* = 48) was vaccinated for other reasons. Among 131 participants immunized preoperatively - 12 (9.2 %) took two doses of vaccine, 99 (75.6 %) – three doses, 16 (12.2 %) - four doses, 4 (3.1 %) did not give any information about the number of doses taken.

### Factors associated with vaccination

Factors associated with positive HBV vaccination status are presented in Table 3 (a bivariate analysis was carried out). Regarding age, significantly less (*p* = 0.04) of those under and equal to 50 years were immunised (73/148; 49.3 %) compared to those between 51 and 60 years of age (77/123; 62.6 %). No significant differences (*p* = 0.36) were found between immunisation rates in those older than 60 years (77/139; 55.4 %) versus those under and equal to 50 years (73/148; 49.3 %), nor between immunisation rates in those older than 60 years versus those between 51 and 60 years old (77/123; 62.6 % vs 77/139; 55.4 %); *p* = 0.29.

**Table 3:** Factors associated with positive vaccination status (bivariate analysis); univariable model results

Variable	Vaccinated	*N*	%	*p*
Age (years) <=50 >50	73	148	49.3	0.08
	154	262	58.7	
Gender: female male	157	271	57.9	0.18
	70	139	50.4	
Residence: urban rural	156	266	58.6	0.09
	71	144	49.3	
Education: university lower	50	73	68.5	0.02
	177	337	52.5	
Socio-economic status: high middle/low	36	55	65.5	0.14
	191	355	53.8	
Employment status: employed not employed	109	193	56.5	0.74
	118	217	54.4	
Type of facility: urban rural	181	336	53.9	0.24
	46	74	62.2	
Past surgery: yes no	181	280	64.6	<0.0001
	45	114	39.5	
Knowledge on HBV: high low	118	192	61.5	0.03
	109	218	50.0	

Zgorzelec county, Poland, 2013 (*n* = 410)

Immunisation rates varied by participants’ literacy with significantly lower rates observed in those with a primary education level (82/185; 44.3 %) comparing with high school graduates (95/152; 62.5 %); *p* = 0.001 and university graduates (50/73; 68.5 %), *p* = 0.0008.

Concerning residency, 58.6 % of those living in the cities were immunized, compared to 49.3 % living in the rural areas; *p* = 0.09.

In those having surgery in the past, immunisation rates were higher than those not having surgery (64.6 % vs 35.5 %), *p* < 0.0001.

The median knowledge score was 16 points. In those more knowledgeable (knowledge level >75 %, i.e. >16.5 points) - 61.5 % were vaccinated, significantly more (*p* = 0.03) than among less knowledgeable ones (50.0 %).

No significant differences were found between immunisation rates regarding gender (*p* = 0.18), socio-economic (p = 0.14) and employment status (*p* = 0.74), as well as facility location (*p* = 0.24).

A multivariable regression model revealed that living in an urban area, and having surgery in the past were each determinants of vaccinating for HBV (Table 4); facility location (urban GP clinics: 4 and 5) was associated with greater odds of poor immunization.

**Table 4:** Logistic regression model: association of HBV vaccination among primary care clinic patients with selected variables (OR’s estimates, 95 % CIs of OR estimates)

Variable / category	OR^a^	95 % CI
Age > 50 years	1.55	0.964-2.512
Gender	0.97	0.605-1.556
Education: university degree	1.95	0.978-4.021
Residence: town	2.11	1.170-3.858
Socio-economic status: high	1.34	0.664-2.894
Surgery in the past	2.73	1.697-4.433
Knowledge on HBV infection: high	1.55	0.983-2.444
Facility location: GP clinic 2	0.90	0.440-1.834
Facility location: GP clinic 3	1.18	0.540-2.567
Facility location: GP clinic 4	0.35	0.171-0.703
Facility location: GP clinic 5	0.40	0.194-0.812

^*a*^ *Odds ratio = vaccination ratio between the two categories tested*

*in each variable, controlling for other variables*

*Zgorzelec county, Poland, n = 410; 2013*

### Anti-HBc total in unvaccinated participants

Out of 183 unvaccinated patients, 7 (3.8 %) refused to give blood. The prevalence of anti-HBc total among unvaccinated participants was 13.6 % (24/176; 95%CI:9.3 %-19,5 %).

Out of 227 vaccinated patients, 33 (14.5 %) refused to give blood. Anti-HBc prevalence in those who agreed was 7.2 % (14/194; 95%CI:4.4 %-11.8 %), 6.3 % (6/95) in vaccinated preoperatively and 8.1 % (8/99) in vaccinated for other reasons.

None of anti-HBc positive participants had the history of clinical HB, none was aware of an infection.

## Discussion

### Overview of the results

This study contributes to the literature by discussing the HBV vaccination uptake among adult patients attending GP practices in the context of immunization determinants and anti-HBc total presence.

The HBV vaccination coverage among adult patients in this study was 55 %, almost one in five of those patients took a booster. The coverage was not satisfactory, however, much higher than in the other countries. More than a half were immunized due to the recommendations of surgeons referring them for various surgical procedures. Sadly, only one in twenty patients was immunized due to the recommendations of a family doctor. Living in a city, as well as having a surgery in the past were each associated with greater odds of being vaccinated. In unvaccinated patients, one in seven presented the evidence of HBV infection; more than 7 % of those vaccinated were anti-HBc total positive.

### HBV vaccination uptake

In Poland, HBV vaccination is offered by a range of healthcare facilities, however primary healthcare settings take the lead, mainly because of their universal access. Therefore, the poor involvement of family doctors regarding HBV immunization observed in this study causes concern and should be a subject of deeper analysis. GPs seem to neglect this very important issue and do not discuss the benefits of HBV vaccination with their patients. It is noteworthy that HCWs involvement has a correlation to the completion of vaccinations [16–18]. However, for many HCWs time may be a barrier to discuss immunization at patient’s visit. In a national US study 56 % pediatricians pointed at that reason [17]. On the other hand, patients indicate they want their primary providers to personally discuss the issue with them [18, 19]. As reported also by others [16, 17, 20], as well as in our previous study [19], media, friends or relatives do not play a role as credible sources of information on vaccination.

The HBV vaccination rate among study participants is similar to that observed in 2011 among patients of the Family Doctor Office and Cardiology Clinic in the Polish city of Katowice [21], and also that observed among 1652 adult surgical patients surveyed in our own previous study, in 2009 [11]; self-reported information was used in both surveys. Of note, the HBV vaccination coverage among surgical and gynecologic nurses from Polish hospitals surveyed by us in 2010 was 100 % [22].

HBV vaccination coverage assessed by a nationwide cross-sectional telephone survey in Germany in 2004, was 30 % [20]. In a more recent study from Hong Kong it was only 26 % among the local Chinese population and 33 % among pregnant women [23, 24]. Vaccination uptake was assessed through face-to-face questionnaire surveys. Among randomly selected Chinese adult immigrants residing in Vancouver, Canada, the vaccination uptake was 38 %; face-to-face interviews were conducted to assess the immunisation rate [25]. Data of men who participated in a nationwide interview survey in the Republic of Korea showed that only 33 % received all three doses of HBV vaccine [26]. The number of doses of HBV vaccine received was based on self-reported information.

### Factors related to HBV immunization

Our results may be useful in developing strategies to increasing HBV vaccination coverage in the adult population not only in Poland, but also in other countries, by identifying the vaccination predictors. Similarly to the results obtained in our previous study [11], the place of residence influenced the vaccination uptake. Participants coming from rural areas may have limited or inconsistent access to healthcare, or limited need for immunization, or their need may go unrecognized by primary care personnel.

The rate at which participants completed the vaccination series increased as a function of age regarding patients in their 50s who were significantly more likely to receive HBV vaccination than were those younger. However, studies from abroad showed that age was inversely associated with adult vaccination status [20, 24, 27], which reflects a quite opposite trend than observed in the present study. The possible explanation of our findings may be that - similarly to those observed in another Polish survey [11] - more than a half of the patients immunized themselves before surgery. Therefore, the detected trend may reflect specific Polish HBV vaccination policies and simply illustrate an increasing need for various surgical procedures among older patients. Further studies are needed to confirm this hypothesis.

A higher level of education was positively associated with the HBV vaccine uptake in the univariate analysis, but not in the logistic regression model, possibly due to the relatively small sample size as well as interactions between variables, e.g. between education and socioeconomic status. Surveys showed that attainment of higher levels of education had a positive effect on HBV vaccination coverage [26–28]. It has been proved that educational level is strongly correlated with the amount of health information sought, including vaccination-related issues [26]. It can influence health-communication behaviors [29] and the acquisition and integration of new information [30].

More than a half of primary care patients in our study had been immunized against HBV due to the recommendations of surgeons, which influenced the overall vaccination uptake. Furthermore, multilevel regression analysis revealed that those having a history of any surgery in the past had a greater odds of being immunized. In addition, most of those completed a 3-dose vaccination course. Therefore, our findings show the effectiveness of the preoperative HBV vaccination strategy and support the rationale for HBV immunisation to surgical patients admitted for elective procedures. It has been suggested [31] that vaccination of newborns together with other polices, targeting adults, should be implemented autonomously, as they complement each other. Preoperative immunisation policy seems to be a significant component of preventive methods, which have an impact on the decrease of HBV incidence rate in the community.

The multivariable regression analysis revealed that patients from two specific GP clinics (both located in urban areas) had lower odds of being immunized. It might be influenced by education strategies experienced by patients which were used by medical personnel in terms of HBV vaccination. Such an association due to organizational differences between different facilities and vaccinators was reported by others [32–34]. Additionally, in the previous study on determinants of self-paid vaccinations in 0-5-year-old children from GP practices in Poland we found that in the group of parents for whom the high price of a vaccine was an important obstacle in a child’s immunization, the strongest determinant for vaccinating a child was the type of facility, which emphases an inevitable role of primary care HCWs in optimal vaccine uptake [19].

As proven by this study, HBV is still a public health threat in Poland, with 14 % of unvaccinated patients and 7 % of those vaccinated presenting serological markers of this infection. Of note, none of those was aware of the condition, being a possible source of infection for others.

However, as HBV core total antibody is a marker of past or current infection, without additional tests it is not possible to determine the year of infection for those participants with evidence of anti-HBc total. Potentially, some of these infections might occur prior to the introduction of preoperative vaccination. According to the data obtained by this sero-survey, anti-HBc prevalence in the individuals vaccinated preoperatively was 6 %. It could be possible that most of those participants were infected with HBV before vaccination and were not tested for the markers of a previous infection before the immunisation procedure [9, 11, 22]. Of note, although the preoperative immunisation policy in Poland required HBsAg checking, it did not refer to anti-HBc. Another explanation is that some participants were vaccinated but did not respond. As the anti-HBs titer was not checked after the vaccination course, they were not aware of the fact they were in the non-responders group. Since they were still vulnerable, they contracted an infection.

Regarding HBV vaccine, the three-dosage regimen is recommended for its effectiveness [1, 5, 35]. Those who develop a protective antibody response are protected from clinical disease and chronic infection. The cost of a course of HBV vaccination for adults in Poland, although entirely covered by patients, is relatively low (around 50$ US). However, one in nine patients in the present study received only two doses of vaccine. Seroprotection rates approach 95 % among healthy adults after receiving the complete course of HBV vaccination [1, 5, 35], but decrease up to 87 % among those who received two doses only [36, 37]. It puts forward the necessity of completing the three-dose vaccination course. Nevertheless, it should be noted that, as presented in Table 2, more than one fourth of those receiving two doses 0–12 months previously might have been due to receive a further dose of vaccine to complete their course of vaccination.

### Limitations

Our results may be not be generalizable to the other GP practices located in other regions of the country, especially in towns of more than 40.000 inhabitants. Further studies on national level would be of value. Secondly, while we highlighted patients’ demographic, other factors might have also influenced vaccination coverage.

The strength of the study was due to the involvement of patients from randomly selected primary care practices, with an excellent response rate of 98 %. Moreover, HBV immunization status was not only based on self-reports of previous immunization but also on the results of vaccination cards and serology tests which are perceived as reliable means of determining the vaccination status of the participants.

Data relating to vaccination status could be collected via face-to-face interview, via vaccination cards and via anti-HBs serology. There are inconsistencies between these data sets indicating vaccination, which should be discussed. The limitation of the studies based on face-to-face interview techniques is the reliability of reported vaccination against HBV, which reportedly gives rise to some inaccuracy [20] (subject to faulty recall); this could lead to both: under- or overestimation of the results. The cross-checking vaccination cards or GP records could possibly be subject to incorrect completion, which could lead to an underestimation of immunization coverage. Finally, assessments of vaccination status based on anti-HBs test results could be biased by the possibility of prior HBV infection and lead to overestimation.

## Conclusions

HBV immunization coverage among adult patients in Poland is not satisfactory, however, much higher than in the other countries. Low HBV immunization coverage among adult patients and the presence of serological markers of HBV infection among both - those unvaccinated and vaccinated, call for comprehensive preventative measures against infection, including greater GP practices involvement. Although interventions should cover the whole population, inhabitants living in the rural areas should be a group of special interest.

The uptake of HBV vaccine is excellent among Polish newborns and teenagers [38], however adults seem to be a neglected group. Thus, vaccination strategies should be more optimized, and should include additional health promotion and education programs implemented on a national scale. We find evidence that recommended pre-operative immunization for HBV is efficient in increasing the vaccination uptake. Hence, it should serve as a supportive public health tool to limit the spread of the epidemic, especially in those countries in which nosocomial HBV transmission remains a common route.

To increase the uptake it may be necessary to develop tailored approaches that take into account the factors identified in this study. Family doctors play a marginal role in HBV vaccination. Hence, future interventions should be addressed especially to GPs, making them crucial players in the battle against HBV. Primary care staff should be aware of their importance in recommending vaccination to those at risk of HBV infection. Patients should be provided with adequate information, advice and support to enhance vaccination coverage which, in the long run, will help to develop herd immunity. The presence of serological markers of HBV infection among one in seven of unvaccinated study participants and among 7 % of those vaccinated stress the need for comprehensive preventative measures on population level.
